# Growth mechanism study and band structure modulation of a manganese doped two-dimensional BlueP-Au network†

**DOI:** 10.1039/d3ra00751k

**Published:** 2023-04-24

**Authors:** Yuxuan Guo, Chen Liu, Jiangwen Liao, Yunpeng Liu, Haijie Qian, Jinfeng Xu, Hao Wang, Kaiqi Nie, Jiaou Wang

**Affiliations:** a Institute of High Energy Physics, Chinese Academy of Sciences Beijing 100049 China wangjo@ihep.ac.cn niekq@ihep.ac.cn guoyuxuan@ihep.ac.cn +86-010-88235992; b University of Chinese Academy of Sciences, Chinese Academy of Sciences Beijing 100049 China

## Abstract

Two-dimensional (2D) materials are a very promising material family. The two-dimensional inorganic metal network called BlueP-Au network is rapidly attracting the attention of researchers due to its customizable architecture, adjustable chemical functions and electronic properties. Herein, manganese (Mn) was successfully doped on a BlueP-Au network for the first time, then the doping mechanism and electronic structure evolution was studied by *in situ* X-ray photoelectron spectroscopy (XPS) based on synchrotron radiation, X-ray absorption spectroscopy (XAS), Scanning Tunneling Microscopy (STM), Density functional theory (DFT), Low-energy electron diffraction (LEED), Angle resolved photoemission spectroscopy (ARPES), *etc.* Mn atoms tend to be stably adsorbed on two sites of the BlueP-Au network. It was the first observation that atoms can absorb on the two sites stably simultaneously. It is different from the previous adsorption models of BlueP-Au networks. The band structure was also successfully modulated, and overall down about 0.25 eV relative to the Fermi edge. It provided a new strategy for customizing the functional structure of the BlueP-Au network, which has provided new insights into monatomic catalysis, energy storage and nano electronic devices.

## Abstract 2

We introduced Mn atoms into the BlueP-Au network for the first time in an experiment and proposed a growth model. We found the band structure was modulated after Mn deposition.

## Graphical abstract

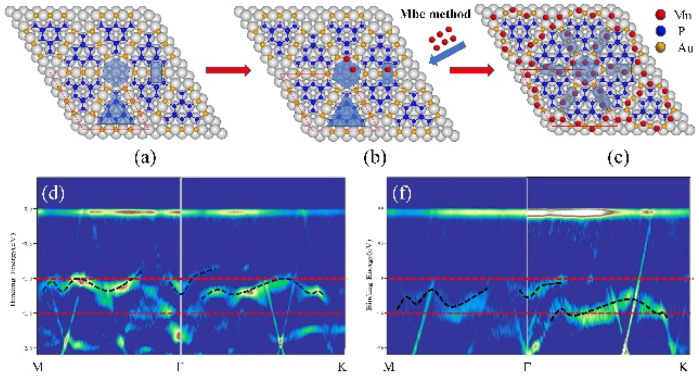

## 1. Introduction

2D materials often have superior properties and exotic physical phenomena,^1–3^ which make them attract the attention of researchers. Due to the thickness of 2D materials, both carrier migration and heat diffusion are confined in the 2D plane, which makes this material exhibit many unique properties, such as Dirac cone,^4^ Quantum anomalous Hall effect^5^ and so on; due to its tunable band gap, it is also employed in field effect transistors, optoelectronic devices, and thermoelectric devices.^6^ Its controllable spin and valley degrees of freedom have also attracted spintronics and valleytronics study.

2D inorganic metal network materials have attracted much attention recently.^7^ They are very interesting and of great potential for researchers to explore. The concept is inspired by porous organic network materials. It is well known that porous organic networks have many new applications in sensing, catalysis, gas storage and topological materials.^8^ Moreover, inorganic materials can withstand more harsh environments and have better electronic properties than organic materials. The concept of module assembly derived from porous organic networks can be applied to construct 2D inorganic porous structures. It will be a very interesting and valuable field of research. So far, the construction and performance modulation of 2D inorganic metal networks are still under exploration, which needs further investigation.^7^

**Fig. 1:**
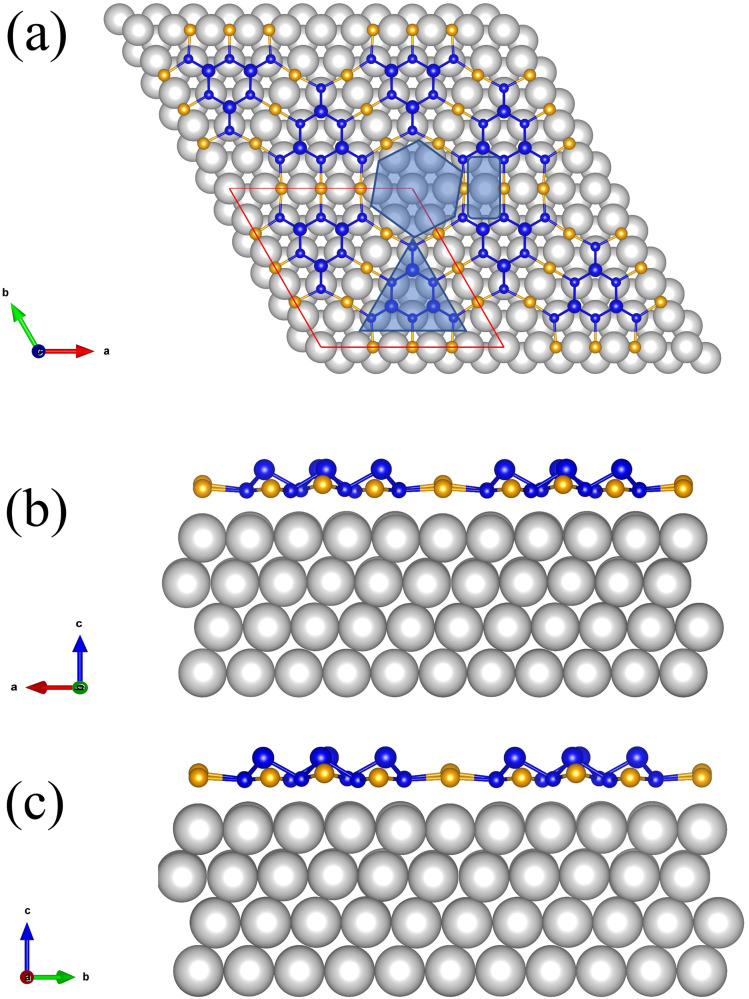
Schematic diagram of BlueP-Au network. The yellow balls represent the Au atoms of the BlueP-Au network layer, the grey balls represent the bulk Au atoms. The blue balls represent P atoms, and the size of the balls represents the P atoms in high position or in low position. The red parallelogram represents the superstructure of BlueP-Au network.

Due to its interesting physical properties, the 2D metal-inorganic network BlueP-Au has attracted the attention of many researchers.^7^ The BlueP-Au network was originally discovered when blue phosphorus (BlueP) was grown on Au(111). In 2016, Chen's group reported that the blue phosphorus structure was grown on the Au(111) plane for the first time,^9^ and its semiconductor properties were measured. Then it was subsequently repeated by multiple research groups.^10,11^ However, in 2020, Tong's research group accurately measured the atomic structure of the so-called BlueP on Au(111). They proposed that the “BlueP structure” in the STM image could not match the theoretical blue phosphorus structure, but rather a triangular blue phosphorus “island” composed of nine P atoms (P9). The islands are connected by three Au atoms on both sides of the island, forming a metal-phosphorus network, called BlueP-Au network.^7^ As reported by the team, a schematic diagram of its structure is shown in Fig. 1(a). The BlueP island (P9) forming a honeycomb structure as marked by a triangle due to its triplet rotational symmetry. The primitive cell of this structure contains 18 P atoms and 9 Au atoms. The unit cell of this structure is matched to the 5 × 5 Au(111) lattice. The network itself has three pore sites, the Hollow site in the middle (Fig. 1(a), hexagonal area), the bridge site (Fig. 1(a), rectangular area) and the BlueP honeycomb site (Fig. 1(a), triangular area of the honeycomb structure P in the middle of the pore site), which are very suitable for the deposition and adsorption of specific elements. The evidence shows that the chemical function and electronic properties of the network can be tuned by changing the connecting atoms, subunit units or adsorbing relevant atoms at specific pore sites.^7^ It will provide an opportunity to design 2D porous networks with target properties.

There have been a few related studies on 2D porous networks so far. It was previously reported that when potassium (K) was doped onto the BlueP-Au network layer, K ions would insert *via* the holes and impair the layer's connection with the substrate.^12^ There are also research reports that doping silicon (Si) on it can remove the interaction between the network and the substrate. Then the BlueP islands were reconstructed and formed freestanding BlueP.^13^ This is the first time to synthesize blue phosphorus. Additional research has focused on depositing organic PTCDA^14^ on it, which results in the generation of an organic film with a herringbone structure. It has also been reported that when tin (Sn) is deposited on BlueP, Sn would preferentially adsorb in the hollow sites and be independent of each other, and Sn single-atom catalysis is successfully realized.^15^ Using the holes of a 2D inorganic network to uniformly and independently load specific atoms to achieve special functions is an ingenious idea. However, there are not much related researches, and the doping on it is often the main group element or organic matter. Research on doping transition metals is quite limited so far.

Transition metal elements tend to have unfilled d orbitals in the valence shells, and their properties are often very different from other elements.^16,17^ Mn, one of the transition metal elements, always plays a significant role in the field of materials. It is easy to bond with other substances because of its 3d orbital activities, and antiferromagnetic substance.^18^ Previous research has demonstrated that the introduction of Mn into 2D materials results in a variety of fascinating occurrences. For example, 2D materials are often difficult to become magnetic. But it can be achieved magnetic by doping Mn, and graphene is a typical example.^19^ Additionally, theoretical calculations demonstrate that Mn will be adsorbed on MoC_2_, producing a magnetic moment without affecting the original structure of MoC_2_.^20^ Additionally, as a luminous ion,^21^ the Mn ion can be used to create composite 2D materials with unique optical properties by mixing it with other 2D materials. For instance, Mn-doped MoSe_2_ can lengthen the material's lifespan of luminescence.^22^ In addition, a study of IOP showed that an anomalous quantum Hall effect appeared after Mn doping Bi_2_Se_3_, and two anomalous quantum Hall effect components were found, which is very interesting.^23^ Theoretical research on Mn-doped 2D materials^24,25^ has made a lot of progress, but more experimental evidence are still needs to be further studied.

In this paper, we attempted to dope the transition metal element Mn into the BlueP-Au network using the MBE method. The whole doping process was studied in detail by *in situ* XPS, XAS, STM, ARPES, LEED and other characterization methods. A possible adsorption doping model of Mn in BlueP-Au network was proposed. It is also found that the electronic structure of the system is obviously modulated by the doping of Mn. This provides a new path for the functional design of 2D inorganic metal networks.

## 2. Methods

The experiments were performed in 4B9B endstation of BSRF. The laboratory is equipped with an MBE chamber, an analysis chamber, and an STM test chamber, which are connected to each other through a transfer chamber. The base pressure of all chambers is better than 5 × 10^−10^ Torr. All experiments are conducted in this ultra-high vacuum interconnected system to ensure that there is no atmospheric interference.

### 2.1 Synthesis of 2D BlueP-Au network

BlueP-Au network was grown in a chamber with a base pressure of 3 × 10^−10^ Torr by MBE method. The substrate is Au(111) single crystal, and the argon gun is focus FDG 15X. The surface of Au(111) single crystal which had been degassed and cleaned by Ar ion bombardment (1 kV, 1 × 10^−7^ Torr, 10 min) followed by annealing at 530 °C for 30 min, and the cycle was repeated several times. In order to build the BlueP-Au network, phosphorus vapor was created by heating and evaporating high-purity black phosphorus bulk in a tantalum boat in the MBE chamber. During the growth process, the substrate temperature was set at 260 °C and the growth time was 20 min.

### 2.2 Doping Mn on 2D BlueP-Au network

The entire doping process is carried out in a chamber with a base pressure of 3 × 10^−10^ Torr. The BlueP-Au network previously grown on Au(111) was used as the substrate. The Mn atomic vapor was obtained by heating solid Mn (99.999% purity) in the beam source furnace equipped with cooling water in the chamber to 680 °C. Then the Mn vapor was deposited on BlueP-Au Network as dopants. The substrate temperature was room temperature and the doping time was 1.5 min.

### 2.3 Characterization of structural and electronic structure

*In situ* XPS, ARPES and LEED characterizations were performed in SCIENTA R4000 analyzer system, and STM scanning was performed in STM system at room temperature. Synchrotron radiation was used as the light source, and the excitation photon energy was 180 eV for P-2p and Au-4f XPS measurement, with an energy resolution of 0.1 eV, at room temperature. The monochromatic He–I light source (21.2 eV) was used for band dispersion measurement in the analysis chamber equipped with a low-temperature cooling system, and the test temperature was 8 K. The total energy resolution was set to 15 meV, the angular resolution was set to ∼0.3°, and the momentum resolution was set to ∼0.01 π/a. STM measurements were performed using a normothermic STM scanning near-field optical microscope system, where a bias was applied to the substrate. The LEED was measured in the analysis chamber with ErLEED-1000A from Specs, and the measured voltage was 79 V, and the current was 2.05 A.

### 2.4 The DFT calculation

We use the Vienna *ab initio* simulation package (VASP) to perform first-principles calculations based on density functional theory (DFT).^26^ The generalized gradient approximation (GGA) with Perdew–Burke–Ernzerhof (PBE)^27^ exchange-correlation function was considered in all calculations. Projected enhanced wave (PAW)^28^ potentials were used to explain electron–ion interactions. A generalized gradient approximation (GGA) based on the Perdew–Burke–Ernzerhof (PBE) scheme was used to explain exchange-related effects. A plane wave basis set with a kinetic energy cutoff of 350 eV was used, and the convergence criteria for electron self-consistent total energy and force for geometric optimization are set to 10^−4^ eV, and 0.05 eV Å^−1^ respectively during all the calculation. We sample the Brillouin zone using 21 × 21 × 21 Monkhorst–Pack *k* points during bulk Au single crystal relaxation. Au(111) surface slab is modeled with four layers of Au atoms, and the bottom two layers of Au atoms remain fixed in their bulk positions, and the other two layers are not fixed, and the thickness of vacuum layer is 20 Å. We sample the Brillouin zone using 21 × 21 × 1 Monkhorst–Pack *k* points during geometric relaxation. The BlueP-Au network layer was modeled on the Au(111) surface slab and the vacuum layer with a thickness of 20 Å was included in the superstructure shown by the red parallelogram in Fig. 1(a). The superstructure is matched to the 5 × 5 Au(111) lattice. We sample the Brillouin zone using 2 × 2 × 1 Monkhorst–Pack *k* points, and the DFT-D3 method was employed to account the van der Waals (vdW) interaction^9^ during geometric relaxation. Then Mn atoms were introduced into specific sites of the superstructure for structural optimization.^29–31^ The DFT + U method was employed, and we set *U* = 4.2 eV, *J* = 1.0 eV.^25,32,33^ We sample the Brillouin zone using 2 × 2 × 1 Monkhorst–Pack *k* points, and the DFT-D3 method was employed during geometric relaxation. The adsorption energy *E*_ad_ was calculated as follows

1 *E*_ad_ = *E*_Mn/BlueP-Au_ − *E*_BlueP-Au_ − *E*_Mn_

here, *E*_Mn/BlueP-Au_, *E*_Mn/BlueP-Au_, *E*_Mn_ are the total energies of the Mn doped BlueP-Au network system after relaxation, the BlueP-Au network after relaxation, one Mn atom after bulk Mn relaxation. VASP kit^34^ was used for data processing.

## 3. Results and discussion

**Fig. 2:**
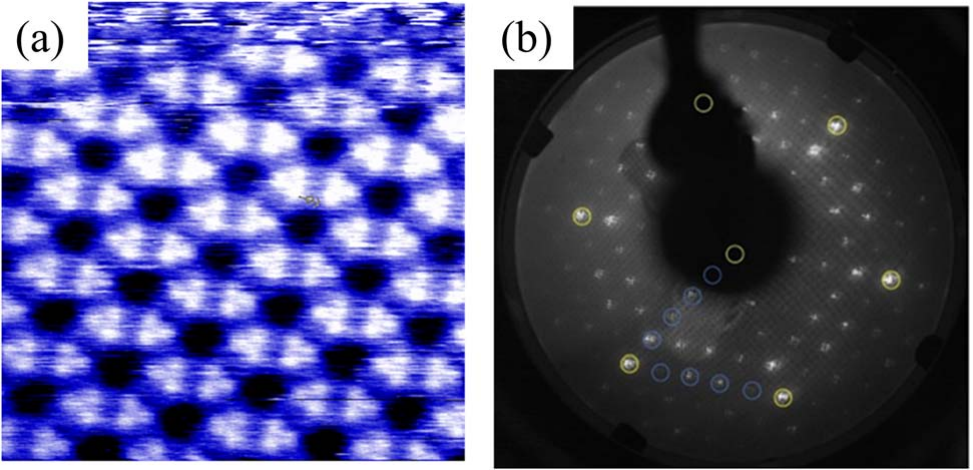
(a) Enlarged STM image of the original BlueP-Au network on Au(111). (b) LEED pattern of BlueP-Au network.

First, we successfully synthesized high-quality BlueP-Au network on the clean Au(111) surface treated by argon etching and annealing cycle by MBE method, which is well revealed by STM and LEED diagrams in Fig. 2. Fig. 2(a) is the STM diagram of BlueP-Au network, in which the regular and repeated hexagonal lattice can be clearly seen. Each hexagon contains six bright spots, and each bright part is the 9-polymer of P. The cell is depicted in the image with a period of about 1.4 nm, which corresponds to about five times of the lattice constant of Au(111) (0.288 nm), and the edges and diagonals of the cell are occupied by three darker atoms. This is basically consistent with the appearance structure reported previously.^12^Fig. 2(b) shows a typical BlueP-Au network diffraction pattern.^12^ The yellow dot corresponds to the Au atom in 1 × 1 Au(111), and the blue dot corresponds to the 4 × 4 P superstructure in BlueP-Au network. The lattice constant of the 4 × 4 P superstructure is exactly five times of Au(111), which is consistent with the previous report.

**Fig. 3:**
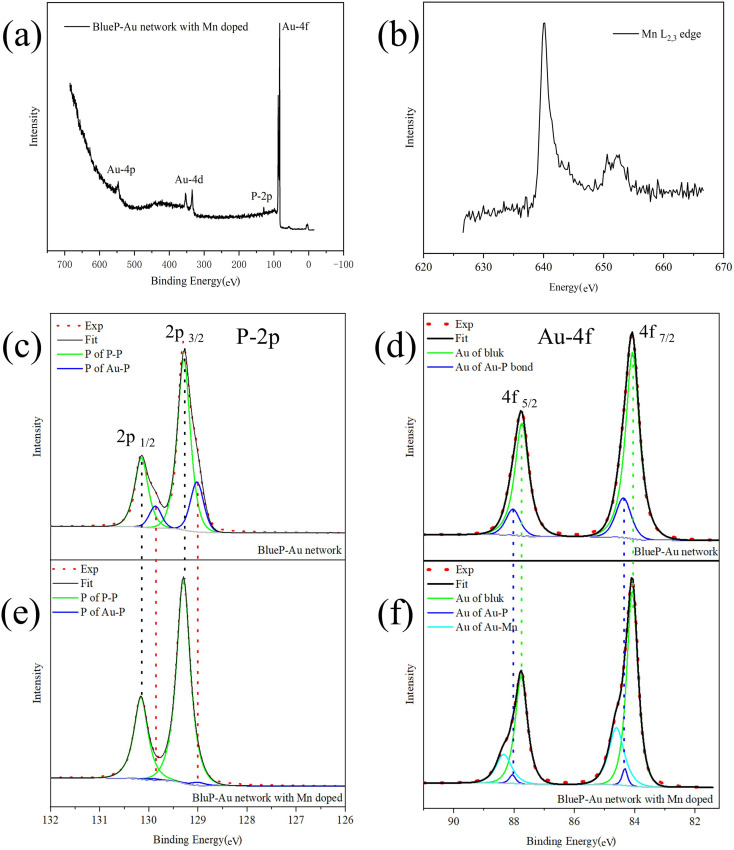
(a) The XPS survey spectrum of BlueP-Au network with Mn-doping (b) the XAS spectral of Mn L_2,3_ edge. (c) and (d) The Au-4f and P-2p XPS spectral of the pure BlueP-Au network, respectively. The red dotted line represents the experimental results, the black solid line represents the fitting results, and the green and dark blue solid lines represent the different components of the same element fitted by peak fitting. (e) and (f) The Au-4f and P-2p XPS spectral lines of the BlueP-Au network doped with Mn, respectively. The red dotted line represents the experimental results, the black solid line represents the fitting results, while the green, dark blue and cyan solid lines represent different components of the same element obtained by peak fitting.

In order to study the process of Mn-doped BlueP-Au network, we conducted *in situ* XPS measurement based on synchrotron light source with detailed analysis for the BlueP-Au network and Mn-doped network respectively. Synchrotron radiation has the advantages of high resolution and adjustable photon energy. Considering the photoionization cross section, 180 eV is selected as excitation photon energy to resolved more spectral line details, which are usually not available with conventional light sources. The experimental spectra are represented by red dotted lines, the background is represented by gray lines, the fitting data is represented by black lines, and the fitting peak components are represented by specific color lines as legend indicated in Fig. 3(c)–(f).

The survey spectrum of Mn-dopped BlueP-Au network, is shown in Fig. 3(a). P and Au related photoemission lines can be found, but no obvious characteristic lines of Mn. The reason for this may be that the doping content of Mn is too low. So we conducted the XAS^35–37^ measurement on Mn L-edge and a clear Mn signal^38^ can be collected as shown in Fig. 3(b) The XAS measurement can only be done at the synchrotron radiation facility due to the energy tunability. P-2p and Au-4f core level spectrum of BlueP-Au network are shown in Fig. 3(c) and (d), respectively. In the two figures, the spin–orbit coupling splitting peaks of P-2p and Au-4f can be clearly observed.

The fitting results show that both spectral lines are composed of two bonding components, which are represented by green lines and dark blue lines, respectively. The 2p_3/2_ peak of the P component represented by the green line in Fig. 3(c) located at 129.28 eV, the 2p_1/2_ peak is located at 130.16 eV, and the doublet separation is 0.88 eV, representing the 2p peak of P in P–P bonds; The 2p_3/2_ peak of the P component represented by the blue line located at 129.02 eV, the 2p_1/2_ peak located at 129.87 eV, and the doublet separation is 0.85 eV, representing the 2p peak of P in Au–P bonds; This is in line with the conclusions reported in the previous research.^13^ The 4f_7/2_ peak of the Au component represented by the green line in Fig. 3(e) is located at 84.08 eV, the 4f_5/2_ peak is located at a binding energy equal to 87.73 eV, and the doublet separation is 3.65 eV, corresponds to the Au atom in the bulk phase of Au(111); The 4f_7/2_ peak of the Au component represented by the blue line located at 84.38 eV, and the 4f_5/2_ peak located at 88.01 eV with a doublet separation of 3.63 eV, representing Au atoms at the Au–P bonding. This is basically consistent with the results reported in the previous research.^12^

Next, XPS was performed on the Mn-doped BlueP-Au network, and the spectra are shown in Fig. 3(e) and (f). In the process of analyzing its core level spectrum, we found an interesting phenomenon: with the doping of Mn, the shoulder peak of the core level spectrum of P on the BlueP-Au network gradually weakened and disappeared finally, and a new set of peaks aroused in Au 4f core level spectrum.

In Fig. 3(e), we can see that the P-2p spectrum of the Mn-doped system still consists of two components, which are also represented by the green line and the dark blue line, respectively. The 2p_3/2_ peak of the P component represented by the green line in Fig. 3(b) located at 129.30 eV, the 2p_1/2_ peak located at 130.15 eV, with a 0.85 eV doublet separation; The 2p_3/2_ peak of the P component represented by the blue line located at 129.01 eV, the 2p_1/2_ peak located at 129.90 eV, with a 0.89 eV doublet separation. By comparing with the core level spectrum of the pure BlueP-Au network after fitting the P-2p XPS peaks within a reasonable error range, we can basically conclude that the 2p peak position of P before and after doping with Mn is basically unchanged. The peak positions of the other core levels are also unchanged. However, the intensity ratio of P-2p in the Au–P bond to P in the P–P bond after Mn doping is greatly reduced compared with the ratio of pure BlueP-Au network. This spectra evidence indicates that the interaction between Au and P is greatly weakened after doping with Mn. In Fig. 3(f), we can see that the core level spectrum of Au-4f contains three components after peak fitting, which are represented by green lines, dark blue lines and light blue lines, respectively. The Au 4f_7/2_ peak is represented by the green line in Fig. 3(f) and located at 84.05 eV, and the 4f_5/2_ peak located at 87.76 eV, with a doublet separation is 3.71 eV; The 4f_7/2_ peak of the Au component represented by the blue line located at 84.35 eV, and the 4f_5/2_ peak located at 88.05 eV, with a 3.70 eV doublet separation. Compared with the pure BlueP-Au network, these two components should correspond to the Au atoms in the bulk phase and the Au atoms of the Au–P interface, respectively. Similarly, it can be found that the peak intensity ratio of Au in Au–P layer to Au in the bulk after Mn doping is significantly lower than that before Mn doping. And it can be seen from Fig. 3(f) that a new set of Au-4f peaks appeared at 84.61 eV and 88.32 eV, with a 3.71 eV doublet separation, represented by the solid line in cyan. This peak appeared after the deposition of Mn, which should originate from the interaction between Mn and Au.

Considering the changes of these core level spectra, combined with the positions of Au and P in Fig. 1, it is suggested that Mn atoms are adsorbed into the bridge site and hollow site of the BlueP-Au network. Compared to previously reported models,^7,15^ our experimental result gives a new adsorption model.

**Fig. 4:**
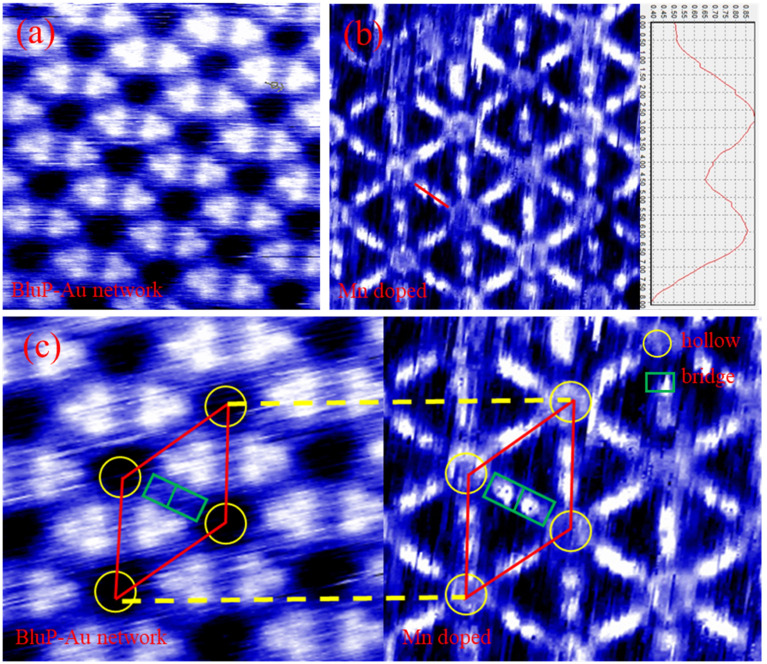
(a) Enlarged STM image of the original BlueP-Au network on Au(111) (*V*_bias_ = 441.9 mV, *I* = 90 pA), (b) amplified STM image after deposition of Mn (*V*_bias_ = 441.9 mV, *I* = 220 pA), (c) it is the schematic diagram of the corresponding primitive cell before and after doping Mn. The red diamond represents the primitive cell, the green rectangle represents the bridge site and the yellow circle represents the hollow site.

Next, we conducted STM tests on the BlueP-Au network before and after Mn doping, as shown in Fig. 4. Fig. 4(a) shows the STM diagram of BlueP-Au network. Fig. 4(b) shows the BlueP-Au network STM diagram after the deposition of Mn. The height map on the right side in Fig. 4(b) corresponds to the red line (*i.e.* bridge site) in the figure. It can be seen from the height map that two Mn atoms are indeed adsorbed on the bridge site. Fig. 4(c) shows the schematic diagram of the corresponding primitive cell before and after doping Mn. The regular and repeated hexagonal lattice can be clearly seen in the diagram, and its primitive cell is marked with a red diamond. The yellow circles represent the hollow sites. The green rectangles represent the bridge sites. We can see that the hollow sites were occupied by atoms after Mn doped BlueP-Au network. It can be clearly seen from the figure that there are two bright spots on the bridge site, which should be the Mn atoms deposited on the bridge site.

This is different from previously reported deposition models for other elements, such as K,^12^ Si,^13^ Sn.^15^ According to previous reports, Si and K tend to penetrate BlueP-Au network, weakening the interaction between BlueP-Au network and bulk Au, and true BlueP is synthesized on Au(111) through Si intercalation. The model given in the article of Sn doping is that Sn can only be deposited stably in hollow sites, but not stably deposited at the bridge sites. In the STM image of Mn deposition we observed, Mn can be stably deposited on the bridge site. This new model of Mn doping which can be deposited stably the bridge sites opens a new idea for the structure modulation of BlueP-Au network.

**Fig. 5:**
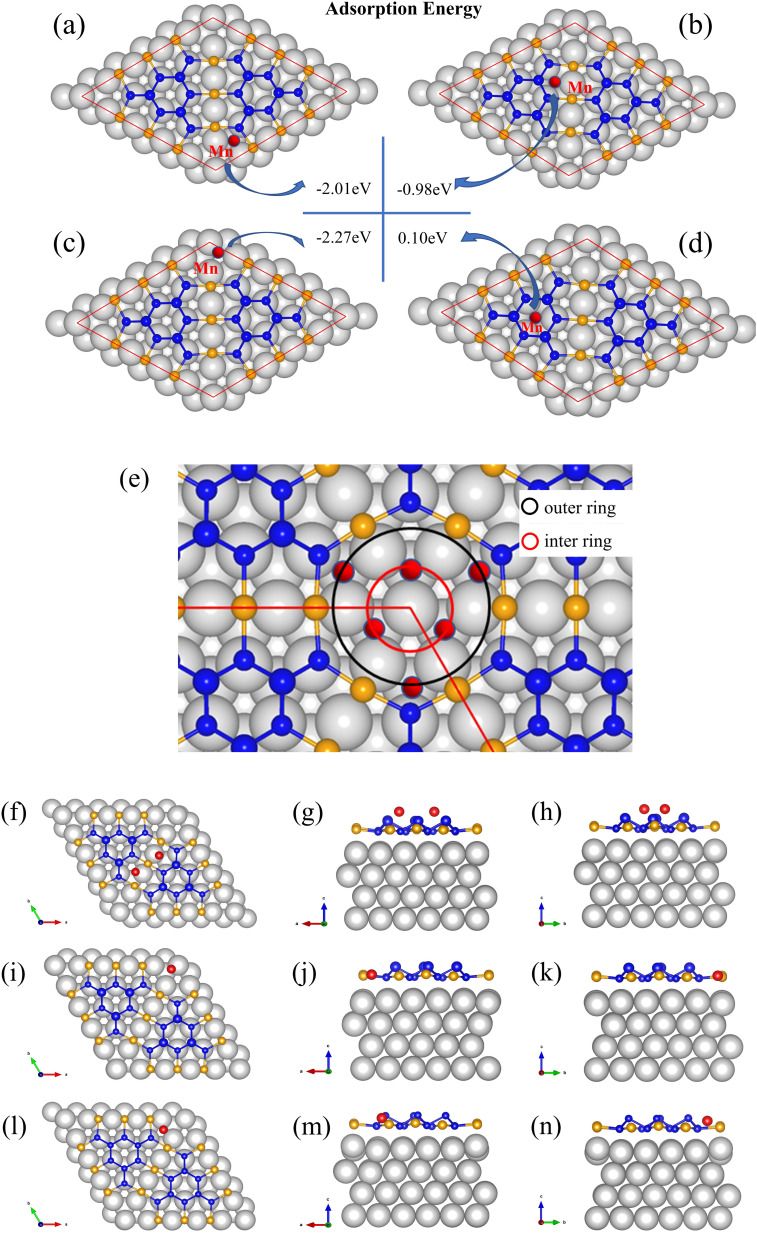
The adsorption energy of Mn on different sites (a) out ring of hollow site, (b) bridge site, (c) inner ring of hollow site, (d) site on BlueP island. (e) The model of Mn adsorption on a hollow site. Optimized structures of Mn on different sites (f)–(h) two Mn atoms on bridge cite, (i)–(k) one Mn atom on inner ring of hollow site, (l)–(n) one Mn atom on outer ring of hollow site.

The first-principle calculations were also performed. The adsorption energies of the four sites on BlueP-Au network were calculated as shown in Fig. 5. By comparing the adsorption energy, it can be found that Mn is easily adsorbed in the inner circle of the hollow cities, as shown in Fig. 5(c). The secondary preferential adsorption is on the outer ring sites of the hollow sites as shown in Fig. 5(a) and bridge sites in Fig. 5(b), and it is difficult to adsorb on the BlueP island in Fig. 5(d). It has been reported that Sn preferentially adsorbs the inner ring and then adsorbs the outer ring in the hollow sites,^15^ and considering the similar radius of Mn atom and Sn atom, the adsorption model of Mn in hollow sites should be similar to Sn adsorption, which adsorbs three positions in the inner ring first, and then adsorbs the other three positions in the outer ring, as shown in Fig. 5(e). As for bridge sites, it can be seen from the Fig. 5(f)–(h) that the optimized model by DFT shows that the bridge site adsorbs two atoms and slightly deviates from the center, which is consistent with the STM results. And the off-center structure is similar to the result of Sn adsorption, though Sn cannot be stably deposited on it.^15^

**Fig. 6:**
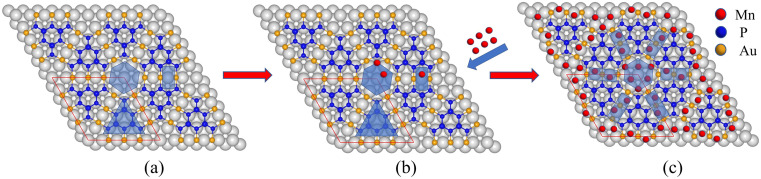
Schematic diagram of Mn adsorption model on BlueP-Au network.

Based on the above experimental result and calculation results, we propose an adsorption model for Mn as shown in Fig. 6. Mn atoms will preferentially adsorb on the inner ring of the hollow site, and then on the outer ring of the hollow site and bridge site. The adsorption model is divided into two parts, the hollow site, and the bridge site. Mn atoms preferentially adsorb the inner ring of the hollow site. After three atoms are adsorbed, they will adsorb on the outer ring of the hollow site. The three Mn atoms in the outer ring of the hollow site will be sitting higher than both the inner ring and also higher than the BlueP-Au layer as shown in Fig. 5(e). That is the same to the Sn atoms.^15^ At the bridge site, the Mn atom will stably adsorb two atoms, as shown in the figure, and is also sitting higher than BlueP-Au layer as shown in Fig. 5(f)–(h). It is the first time to observe that atoms could be stably adsorbed on the bridge sites. Mn atoms are separated independently by the grid and sit higher than the BlueP-Au network layer, which will make Mn atoms independent of each other, and increase the specific surface area of Mn, which is very helpful to promote the study of Mn single-atom catalysis.

**Fig. 7:**
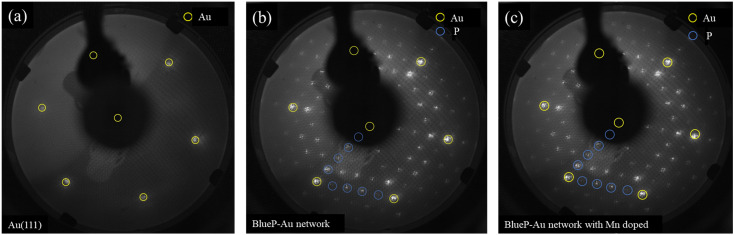
**(**a) Clean Au(111) LEED pattern. (b) BlueP-Au network LEED pattern. (c) Mn doped BlueP-Au LEED pattern. The three LEED patterns were measured at 79 V, and the yellow circle represents the diffraction spot of Au atom of 1 × 1 Au(111), and the blue circle represents the diffraction spot of 4 × 4 P superstructure.

LEED test is also conducted to further evaluate its structure, as shown in Fig. 7. Fig. 7(a) reveals a typical LEED pattern of a clean Au(111) surface with six sharp diffraction spots arranged in a hexagonal pattern. Fig. 7(b) reveals typical BlueP-Au network diffraction spots. Fig. 7(c) shows the LEED pattern of the BlueP-Au atoms after doping with Mn. It can be seen from the figure that the diffraction spots are almost identical to those before doping with Mn, which indicates that the doped Mn atoms did not break the original structure of BlueP-Au network and did not introduce a new periodic structure different from 5 times of Au(111) lattice constant.

**Fig. 8:**
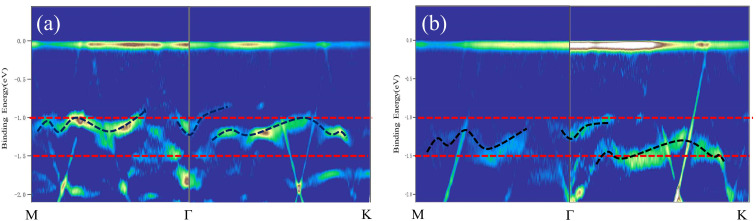
(a) ARPES spectrum of pure BlueP-Au network (b) ARPES spectrum of BlueP-Au network after Mn deposition. Both ARPES spectra are along *M*–*Γ*–*K* direction, and all are treated by secondary differentiation. The white dotted line represents the band folding sp band of Au(111) substrate. The red dotted lines are the reference lines, and the black dotted lines show the main energy bands of the pure and Mn-doped BlueP-Au network respectively.

ARPES measurements collected on BlueP-Au network before and after Mn-doping are to explore its band structure in detail, as shown in Fig. 8, which is used to reveal its energy-momentum dispersion, and then resolved the changes in electronic properties before and after Mn doping. The V-shape band (indicated by the white dashed line) is derived from the folded sp-band of the Au(111) substrate, which has been studied in previous reports.^11,12^ Except for the foldable sp band, the valence band of the original BlueP-Au network marked by the black dashed line is located around 1.0 eV below the Fermi level in Fig. 8(a). This is consistent with previous experiment reports and also consist with previous theoretical studies on the bands of the BlueP-Au network.^13,39^ In the ARPES spectrum of the Mn-doped BlueP-Au network, shown in Fig. 8(b), a significant decrease in the binding energy of this valence band is found, indicating a modulated electronic structure. It should be noted that the modulation of the electronic structure by Mn doping is the result of the electronic doping effect and the weakening of the interaction between Au and P in the BlueP-Au network, which leads to an overall decrease which is about 0.25 eV in the band structure, without any change in band dispersion shape. This also echoes the previous LEED results, that Mn doping did not destroy the original overall structure. Therefore, all these results demonstrate the successful adsorption of Mn atoms and the subsequent modulation of the electronic structure of the BlueP-Au network, revealing the potential applications of this novel 2D inorganic network material in high-performance microelectronic devices.

## 4. Conclusion

In conclusion, we introduced transition metal Mn atoms onto the BlueP-Au network and successfully synthesized a specific structure for the first time in experiment. There is no related experimental research on Mn-doped BlueP-Au network before in experiment. We found that element (Mn) could be adsorbed on both the BlueP-Au network bridge site and hollow site stably simultaneously. This phenomenon was not found in the previous researches on BlueP-Au network doped with other elements. We studied the deposition process of Mn in BlueP-Au network, and proposed a model to explain the process. Mn atoms are preferentially adsorbed on the inner ring of the hollow site, then will be adsorbed on the outer ring of the hollow site, and then two Mn atoms will be stably adsorbed at the bridge site. We also explored the changes in the electronic structure of BlueP-Au network before and after Mn deposition. We found the band structure overall down about 0.25 eV relative to the Fermi edge after Mn deposition. With its distinctive structure, the BlueP-Au network on Au(111) offers some good locations for the adsorption doping of particular elements. The deposition mechanism of Mn on BlueP-Au network provides a new approach and understanding for designing 2D materials with unique functions through doping, such as single-atom catalysis with higher efficiency. It also provides a new method for the combination of transition metal magnetic elements and 2D materials and broadens the application of BlueP-Au network. The deposited Mn weakened the P–Au interaction by bonding to Au. Mn-adsorbed BlueP-Au network can renew the electronic properties of BlueP-Au network by the effects of electron doping and weakening the Au–P bond interaction. The results also provide a possible approach to design the electronic structure of BlueP-Au Network by atomic manipulation, which has broad application prospects in energy storage and nanoelectronics devices.

## Conflicts of interest

The authors declare no competing financial interest.

## Supplementary Material

RA-013-D3RA00751K-s001
