# Supplementary material for: Growth mechanism study and band structure modulation of a manganese doped two-dimensional BlueP-Au network
Source: RSC Adv. 2023 Apr 24;13(19):12685–94. doi: 10.1039/d3ra00751k (PMC10123488; doi:10.1039/d3ra00751k)
Supplement: RA-013-D3RA00751K-s001 [file RA-013-D3RA00751K-s001.pdf]

Electronic Supplementary Material (ESI) for RSC Advances.
This journal is © The Royal Society of Chemistry 2023

Supporting Information

Growth mechanism study and band structure modulation of Manganese doped two-dimensional BlueP-Au network

Yuxuan Guo[ab], Chen Liu[a], Jiangwen Liao [ab], Yunpeng Liu[a], Haijie Qian[a], Jinfeng Xu[ab], Hao Wang[ab], Kaiqi Nie*[,a], Jiaou Wang*[,a]

[a]Institute of High Energy Physics, Chinese Academy of Sciences, Beijing 100049, China

[b]University of Chinese Academy of Sciences, Chinese Academy of Sciences, Beijing 100049, China

***: Correspondence to:** Jiaou Wang, E-mail: wangjo@ihep.ac.cn
Kaiqi Nie, E-mail: niekq@ihep.ac.cn

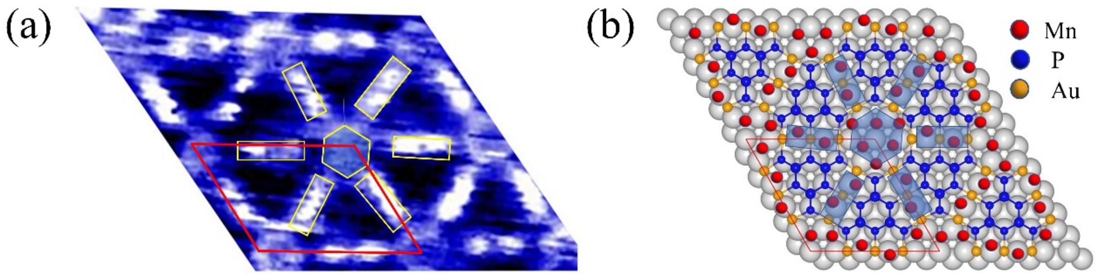

Figure S1. (a) The experimental STM image of Mn doped BlueP-Au network. (b) The simulated STM image of Mn doped BlueP-Au network.

The red diamond represents the primitive cell of Mn doped BlueP-Au network, the rectangle represents the bridge site and the hexagon represents the hollow site. By comparing Fig. S1(a) and Fig. S1(b), we can see that they are a good match.

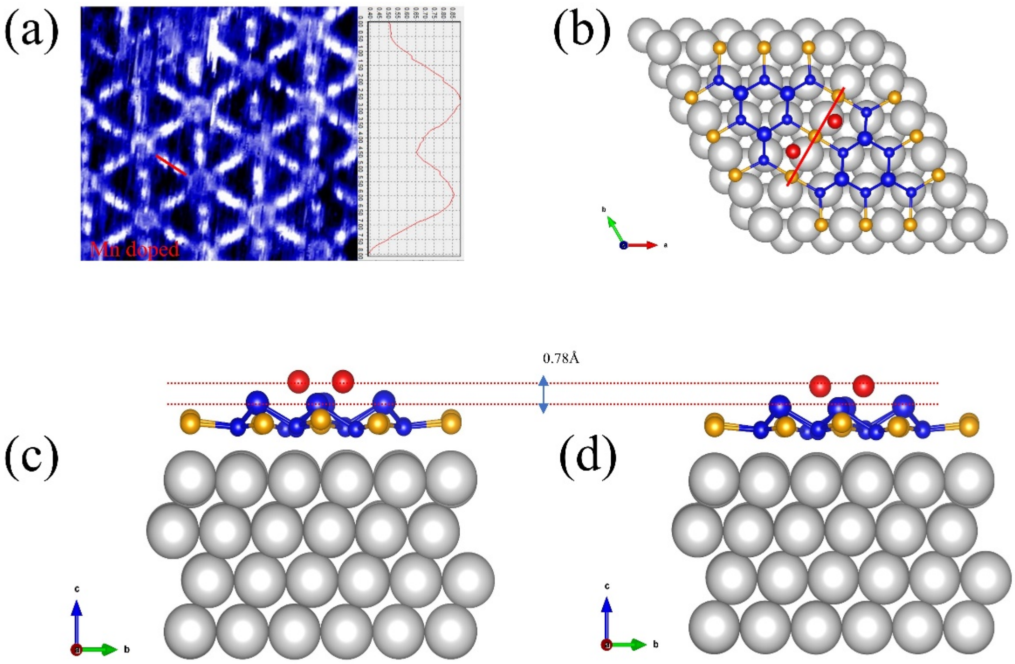

Figure S2. (a) The experimental STM image of Mn doped BlueP-Au network. The red line indicates the height of the adsorbed Mn atoms at the bridge sites. (b) The Front view of the simulated STM image of Mn doped BlueP-Au network. The red line indicates the height of the adsorbed Mn atoms tested at this position. (c) and (d) The side views of the simulated STM image of Mn doped BlueP-Au network. The red line indicates the height of the adsorbed Mn atoms.

From Fig. S2(a), we can know the height of the adsorbed Mn atoms at the bridge sites is 0.45 Å. From Fig. S2(c) and S2(d), we can know the height of the adsorbed Mn atoms at the bridge sites is 0.78 Å. Considering the test error range and model problems, the difference is acceptable. Due to the limitation of STM, the experimental height of the Mn atoms in the hollow sites cannot be obtained. Here, we pay more attention to the Mn atoms adsorbed on the bridge sites, because previous studies have not found that the relevant atoms can be stably adsorbed on the bridge sites to form a specific structure.
